# Population-based differences in cancer incidence between immigrants and non-immigrants in Canada between 1992 and 2015

**DOI:** 10.1186/s12889-025-23117-0

**Published:** 2025-05-19

**Authors:** Hadassah Abraham, Larine Sluggett, Dezene Huber, Robert Olson

**Affiliations:** 1 BC Cancer- Prince George, Prince George, BC Canada; 2 School of Health Sciences, University of Northern, Prince George, BC Canada; 3 Faculty of Environment, University of Northern, Prince George, BC Canada; 4 Department of Surgery, University of British Columbia, Vancouver, BC Canada

**Keywords:** Cancer incidence, Immigrant health, Healthy immigrant effect, Cancer outcomes, Canadian healthcare

## Abstract

**Objectives:**

With increasing immigration in Canada and strained cancer treatment infrastructure, there’s a pressing need for long-term data on immigrant health and cancer incidence. This information is crucial for planning future cancer services and to alleviate the burden on both the population and healthcare system.

**Methods:**

Statistics Canada data were linked from the 1991 Canadian Census, Canadian Cancer Registry, and Canadian Vital Statistics Database to follow a cohort from 1992 to 2015 and compare cancer incidence between immigrants and the Canadian-born for any cancer and specific types of cancers. Immigrants were further classified based on time spent in Canada.

**Results:**

Immigrants had lower odds of developing any cancer (OR = 0.92, 95% CI [0.92–0.93], *p* < 0.001) compared to non-immigrants. However, for stomach cancer and non-cervical gynecological cancers, the odds of cancer incidence were greater for immigrants than for the Canadian-born. Cox regression showed that recent immigrants (0–4 years in Canada) had a lower hazard ratio (HR = 0.77, 95% CI [0.71–0.84], *p* < 0.001) compared to non-immigrants. Those who lived 5–9 years and 10–19 years in Canada had a higher hazard ratio (HR = 0.82, 95% CI [0.75–0.89], *p* < 0.001; HR = 0.90, 95% CI [0.82–0.98], *p* = 0.011), respectively. Immigrants who had been in Canada for 20 years or longer had the highest hazard ratio (HR = 0.98, 95% CI [0.90–1.07], *p* = 0.632), indicating that the so-called “healthy immigrant effect” lessens over time.

**Conclusion:**

Results demonstrated the healthy immigrant effect lessens over time spent in Canada. However, this effect was not uniform across countries of origin and cancer types. Therefore, this research, provides a deeper understanding of immigrant cancer outcomes and will be useful for cancer planning services and cancer control strategies.

**Supplementary Information:**

The online version contains supplementary material available at 10.1186/s12889-025-23117-0.

## Background

The healthcare experiences of individuals in Canada differ depending on immigrant status. Studies have demonstrated a disparity in cancer screening uptake and cancer outcomes between immigrants and non-immigrants due to barriers such as linguistic challenges, cultural distinctions, and lack of information on accessing health services [1–3]. However, studies have also shown immigrants may have better health outcomes, such as lower cancer incidence than non-immigrants, due to the presence of the so-called “healthy immigrant effect (HIE)”. The HIE hypothesis states that recent immigrants arrive in better health than non-immigrants in the host country, having fewer chronic conditions and higher self-reported health. Yet, these rates often converge to that of the host country after a period of time [4]. Researchers have proposed various reasons for this phenomenon, such as protective lifestyle behaviours brought from the home country or, conversely, barriers to accessing healthcare services and a lack of understanding of the healthcare system [5–8].

While international and Canadian studies have demonstrated the presence of the HIE, the phenomenon itself is complex [5, 6, 9, 10]. The apparent benefit of the effect may be misleading and needs further investigation to inform clinical practice and ensure disparities in cancer outcomes between demographic groups are recognized and reduced. As Canada’s population is rapidly diversifying and expanding with immigration, it is crucial to develop a better understanding of the impact of region of birth and duration of residence in Canada on cancer incidence. Certain cancers may pose higher risk depending on the interconnectedness of lifestyle, cultural factors and familiarity with the healthcare system. This knowledge will be beneficial to provide improved patient-centered care and inform policy decisions involving screening strategies and planning of healthcare services.

To our knowledge, there is less evidence on how overall cancer incidence in Canada is impacted by immigrants’ region of birth and how incidence risk changes over a period of 20 years or more, given the HIE may differ based on time spent in the country. It is also less clear how the HIE differs across various cancer types. Thus, we used a population-based study following a large cohort over 23 years using Statistics Canada data to investigate if there are any differences in cancer incidence between immigrants and non-immigrants in Canada, and if so, to gain insight into reasons for these differences and reasons for the apparent HIE.

## Methods

This study used Statistics Canada’s Canadian Census Health and Environment Cohort (CanCHEC), a data linkage including the 1991 long form Census, the Canadian Cancer Registry (CCR) and the Canadian Vital Statistics Database (CVSD). Data were accessed at the Research Data Centre at the University of Northern British Columbia. Ethics approval was received from University of British Columbia’s REB (H21-00373). This population-based retrospective cohort was followed from 1992 to 2015 to analyze cancer incidence between immigrants and non-immigrants.

The CanCHEC cohort was derived from the 1991 Canadian long form Census. The long form Census is a 20% sample of Canadian residents and captures sociodemographic information. Approximately 3.43% of the population were missed–likely comprised of young people, those with low income, highly mobile individuals, unhomed people, and those of Indigenous ancestry [11]. The final CanCHEC cohort included residents of Canada who completed the long form Census questionnaire and were 25 years of age and over as of Census day (June 4, 1991) (3,576,487 individuals). Tjepkema et al. (2019) provide a detailed description of the linkage in their paper [12]. Probabilistic linkage techniques were used to match people to the Canadian Mortality Database and CCR [11].

Variables selected from the Census included self-reported age, sex, total household income, marital status, knowledge of Canada’s official languages (English and/or French), highest level of household education, region of birth, and immigrant status. Region of birth was categorized based on geography and on the United Nations’ World Economic Situation and Prospects (WESP) system [13]. Those in the ‘Other’ category were excluded as they would not provide valuable information in the analyses. Immigrant status was defined based on region of birth– born in Canada (non-immigrants) or born outside of Canada (immigrants). Non-permanent residents (including visitors and persons with work or student visas) were excluded from the analysis as there would be no indication if they immigrated to Canada at a later date for permanent stay. Variables from the CCR included date of cancer diagnosis and tumor classification. Cancers were categorized according to the Surveillance, Epidemiology, and End Results Program (SEER) site grouping variable from the CCR, that grouped cancers according to International Classification of Diseases (ICD)-03 topography and/or ICD-0-3 histology. The CVSD provided the date of death that was used to exclude individuals who died prior to 1992, and for the time-to-event analysis.

To assess the relationship between immigrant status and cancer incidence, multivariate logistic regression and Cox proportional hazards regression models were implemented using Stata 15 statistical software. Models incorporated potential confounders including age, sex, total household income, education, marital status, knowledge of Canada’s official languages and region of birth. Sampling weights were utilized to ensure the cohort was representative of the Canadian population, and bootstrap weights were incorporated to calculate variance estimations. Estimates were rounded according to Statistics Canada requirements.

Multivariate logistic regression models were used to assess the relationship between immigrant status and overall cancer incidence, as well as specific cancers. Pearson and deviance residuals were obtained for the models. The identified outliers did not influence the models and were thus kept in the dataset.

For the time-to-event analyses, immigrants were divided into subgroups based on time spent in Canada, including non immigrants, 0–4 years, 5–9 years, 10–19 years, and 20 + years. Length of time spent in Canada was calculated based on how long an individual had resided in Canada using the Census date (04 June 1991) and date of immigration reported by the individual in the Census questionnaire (ex. 04 June 1991–18 March 1985 = 2269 days divided by 365.25 = 6.21 years). Individuals who died due to any cause were censored. Individuals without immigration dates were classified as non-immigrants.

A Kaplan Meier life table was used to generate an unadjusted and unweighted cumulative incidence graph and included in the Supplementary Material. Due to Statistics Canada vetting rules, the weighted Kaplan Meier graph was not releasable. For the multivariate Cox proportional hazards models, Schoenfeld residuals were used to test assumptions for the continuous variables, and based on the uniform patterns, the assumption was met. The Cox regression models in addition to the logistic regression models were weighted to represent the Canadian population. Descriptive statistics were computed using weighted Chi-square analysis to produce weighted frequencies.

## Results

### Demographic characteristics of the cohort

The total cohort consisted of 17,002,565 individuals (weighted) (Table 1). Compared to the Canadian-born, immigrants were more highly educated, wealthier, older, and were more likely to be either married or single, vs. divorced, widowed, or separated. Immigrants also had a higher incidence of cancer diagnosis (16.8%) compared to non-immigrants (15.9%). The largest difference in cancer-specific incidence rates between immigrants and non-immigrants were demonstrated for lung (2.2% vs. 2.6%) and prostate cancer (5.6% vs. 4.6%).

**Table 1:** Characteristics of the cohort at time of census (results presented as percentages)

Demographics	Immigrant *N* = 3,676,810	Non-immigrant *N* = 13,325,755	*p*-value
**Sex (%)**			< 0.001
Male	48.6	48.4	
Female	51.4	51.6	
**Age (** ***M)***	49.5	45.8	*<.*001
**Highest degree of education (%)**			< 0.001
None	39.5	39.2	
High school	18.2	21.5	
Diploma, college certificate	26.7	27.2	
Bachelor’s or above	15.7	12.0	
**Income (quintiles) (%)**			< 0.001
Q1 < 23,183	21.0	21.4	
Q2 23,184 − 39,152	19.0	21.0	
Q3 39,153 − 54,269	18.0	20.1	
Q4 54,270 − 75,000	19.0	19.4	
Q5 > 75,000	23.0	18.1	
**Marital status (%)**			< 0.001
Single	10.9	19.0	
Married	72.7	62.6	
Separated	2.9	3.4	
Divorced	5.3	7.8	
Widowed	8.2	7.2	
**First official language (%)**			< 0.001
Knows English and/or French	94.0	99.9	
Neither English nor French	6.0	0.1	
**Cancer incidence (%)**			
**Any cancer (%)**	16.8	15.9	< 0.001
Breast	2.2	2.2	0.763
Colorectal	2.2	2.0	< 0.001
Lung	2.2	2.6	< 0.001
Prostate (males only)	5.6	4.6	< 0.001
Head and Neck	0.3	0.4	< 0.001
Stomach	0.5	0.3	< 0.001
Non-cervix gynecological (females only)	1.6	1.5	< 0.001

Note: *M* = mean

Table 2 shows the distribution of immigrants according to region of birth with most immigrants born in Europe, followed by Southeast Asia, followed by reasonably similar proportions for other regions. Immigrants born in Africa and Oceania represented the two smallest cohorts.

**Table 2:** Distribution of immigrants according to region of birth

Region of birth	*N* = 3,676,810 (%)
Europe	58.5
Southeast Asia	14.4
United States (US)	5.8
South Asia	5.1
Caribbean	4.9
Central and South America	4.2
North Africa and Middle East	4.0
Africa	2.3
Oceania	0.8

### Time-to-event analyses

The results of multivariate Cox regression models (Table 3) indicated that the hazard ratio (HR) for cancer incidence increased for every age increase of one year and was also higher for males in comparison to females. Those with a lack of knowledge of either of Canada’s official languages had a lower hazard ratio than those with knowledge of at least one of the languages while those with higher education also experienced a protective effect compared to those with no education. Widowers had a similar hazard ratio compared to single individuals while married, divorced or separated individuals had a higher hazard ratio. After controlling for covariates, the hazard ratios for cancer incidence were lower for immigrants compared to those born in Canada. The unadjusted and unweighted cumulative incidence curves (Supplementary Fig. 1) show a trend of higher incidence of cancer in conjunction with increased time spent in Canada. As in the cumulative incidence curves, a pattern of increasing hazard ratios with additional time spent in Canada was observed (Table 3). Immigrants from Europe and USA had similar hazard ratios compared to Canadian born, while being from other regions of the world had a protective effect. Oceania was automatically omitted from the analysis by STATA as the HR was the same as that of the reference group (Canada).

**Table 3:** Hazard ratios and 95% confidence intervals for cancer incidence among immigrants and non-immigrants in Canada

Factors	HR (95% confidence interval (CI))	*p*-val
**Age**	1.06 (1.06–1.06)	< 0.001
**Sex**		
Female	1.00	
Male	1.39 (1.38–1.40)	< 0.001
**Length of time spent in Canada**		
Non-immigrants (*N* = 13,325,750)	1.00	
Immigrants (0–4 years, *N* = 445,565)	0.77 (0.71–0.84)	< 0.001
Immigrants (5–9 years, *N* = 288,195)	0.82 (0.75–0.89)	< 0.001
Immigrants (10–19 years, *N* = 752,025)	0.90 (0.82–0.98)	0.011
Immigrants (20 + years, *N* = 2,147,670)	0.98 (0.90–1.07)	0.632
**Region of birth**		
Canada	1.00	
US	0.95 (0.87–1.04)	0.262
South Asia	0.65 (0.59–0.72)	<0.001
Southeast Asia	0.82 (0.75–0.89)	< 0.001
Europe	0.99 (0.91–1.08)	0.831
Africa	0.90 (0.82-1.00)	0.040
North Africa and Middle East	0.77 (0.70–0.85)	< 0.001
Caribbean	0.87 (0.79–0.95)	0.003
Central and South America	0.74 (0.68–0.82)	< 0.001
**Language**		
Knows English and/or French	1.00	
Neither English nor French	0.84 (0.81–0.86)	< 0.001
**Total household income**	1.00 (1.00–1.00)	< 0.001
**Education**		
None	1.00	
High school	0.93 (0.92–0.93)	<0.001
Diploma, college certificate	0.93 (0.93–0.94)	< 0.001
Bachelor’s degree or above	0.86 (0.85–0.87)	< 0.001
**Marital status**		
Single	1.00	
Married	1.19 (1.17–1.20)	< 0.001
Separated	1.32 (1.29–1.35)	< 0.001
Divorced	1.35 (1.33–1.38)	< 0.001
Widowed	0.95 (0.93–0.97)	< 0.001

Note: US: United States, CI: confidence interval, p-val: *p*-value

**Fig. 1:**
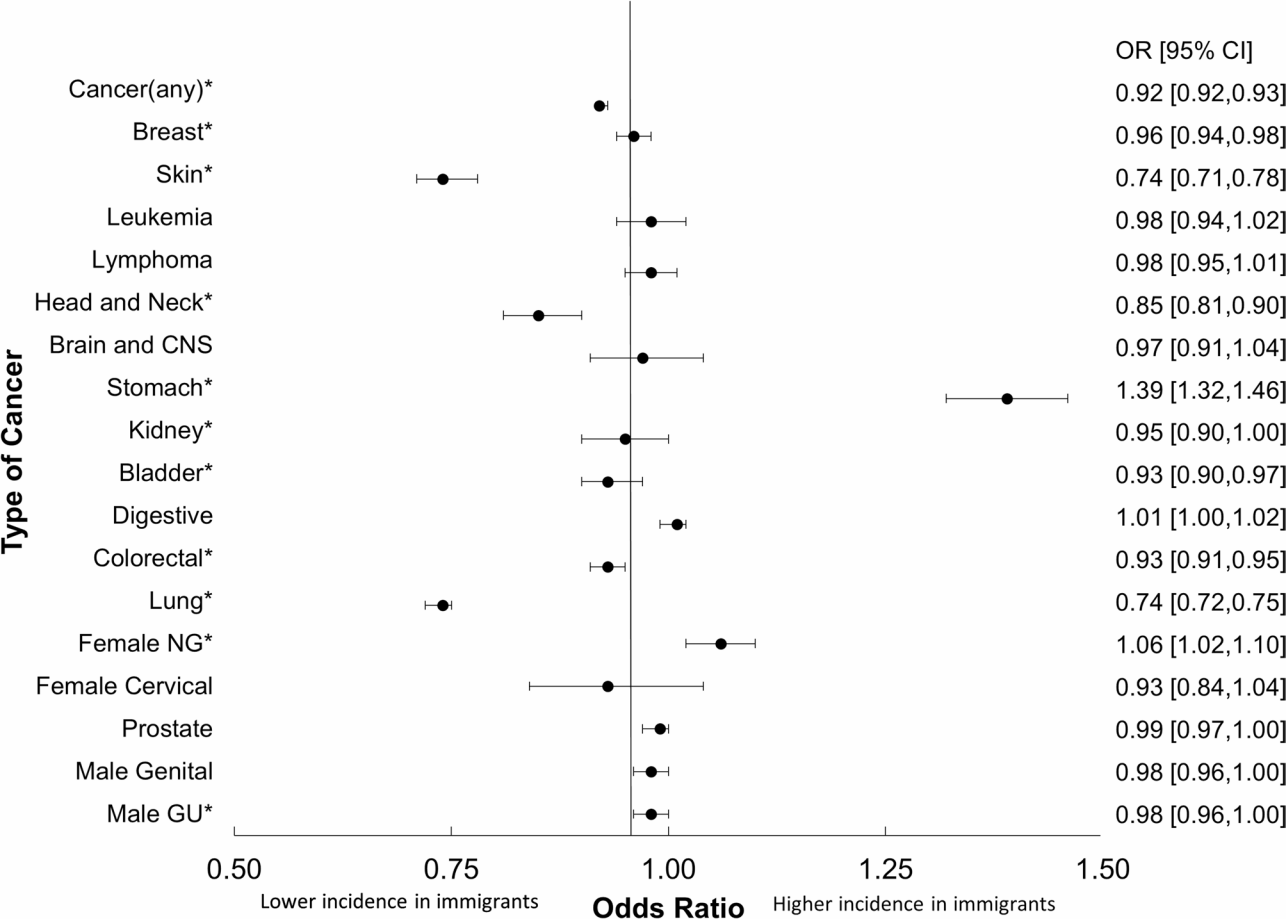
Forest plot displaying adjusted odds ratios of cancer incidence. Note: *statistically significant (α = 0.05), CI: confidence interval, NG: non-cervical gynecological, GU: genitourinary

Immigrants had lower odds of developing any type of cancer [OR = 0.92, 95% CI (0.92–0.93), *p* < 0.001] compared to non-immigrants (Fig. 1). This trend was also observed for other types of cancers except for stomach cancer [OR = 1.39, 95% CI (1.32, 1.46), *p* < 0.001] and non-cervical gynecological cancers in females [OR = 1.06; 95% CI (1.02–1.10); *p* = 0.004].

Table 4 shows the fully adjusted odds ratios for non-cervix gynecological cancers that include vaginal, uterine, and ovarian cancers as well as stomach cancer, while controlling for sociodemographic covariates. For stomach cancer, those born in the US had significantly lower odds of developing cancer while those born in Southeast Asia, Europe, Caribbean and Central and South American countries had higher odds in comparison to the Canadian-born. In females, those born in Europe and Africa had higher odds of developing non-cervix gynecological cancers compared to non-immigrants.

**Table 4:** Logistic regression model with fully adjusted odds ratios for stomach cancer and non-cervix gynecological cancers

	Stomach			Non-cervical gynecological cancer		
	OR	95% CI	*p*-val	OR	95% CI	*p*-val
**Age**	1.04	1.04–1.04	< 0.001	1.02	1.02–1.02	< 0.001
**Sex**						
Female	1.00	-	-	-	-	-
Male	2.04	1.95–2.14	< 0.001	-	-	-
**Region of birth**						
Canada	1.00	-	-	1.00	-	-
US	0.73	0.57–0.92	0.008	0.91	0.80–1.03	0.150
South Asia	0.84	0.62–1.14	0.256	1.04	0.89–1.22	0.617
Southeast Asia	1.63	1.43–1.86	< 0.001	0.89	0.80–0.98	0.019
Europe	1.45	1.37–1.53	< 0.001	1.15	1.10–1.20	< 0.001
Africa	0.98	0.67–1.42	0.907	1.32	1.07–1.63	0.010
North Africa & Middle East	1.03	0.74–1.43	0.853	0.78	0.61–0.99	0.040
Caribbean	1.77	1.42–2.21	< 0.001	0.83	0.70–0.98	0.030
Central & South America	1.57	1.17–2.10	0.002	0.88	0.71–1.08	0.204
Oceania	0.78	0.34–1.74	0.533	0.89	0.60–1.31	0.551
**Language**						
Knows English and/or French	1.00	-	-	1.00	-	-
Neither English nor French	1.13	0.99–1.30	0.079	0.79	0.69–0.91	0.001
**Education**						
None	1.00	-	-	1.00	-	-
High School	0.78	0.73–0.83	< 0.001	1.09	1.05–1.14	< 0.001
Diploma, certificate or college certificate	0.79	0.75–0.84	< 0.001	1.12	1.08–1.17	< 0.001
Bachelors or above	0.60	0.55–0.66	< 0.001	1.19	1.13–1.25	< 0.001
**Income (per $10**,**000)**	1.00	0.99-1.00	0.456	1.00	1.00-1.01	< 0.001
**Marital status**						
Single	1.00	-	-	1.00	-	-
Married	1.30	1.19–1.43	< 0.001	0.99	0.94–1.03	0.547
Separated	1.19	1.00-1.41	0.051	0.84	0.76–0.93	0.001
Divorced	1.23	1.08–1.39	0.001	1.00	0.94–1.07	0.908
Widowed	1.13	1.01–1.28	0.041	0.63	0.59–0.67	*<.*001
**Weighted observations**	17 million			8.8 million		
**# cancer cases**	55,040			131,945		

Note: CI: Confidence interval, *p*-val: *p*-value, OR: odds ratio, US: United States

## Discussion

This population-based study using robust Statistics Canada data showed that immigrants have lower odds of being diagnosed with cancer than Canadian-born people. Immigrants had higher unadjusted crude cancer incidence compared to non-immigrants (16.8% vs. 15.9%), respectively. However, after controlling for socioeconomic and demographic covariates, the models showed that the odds of any cancer incidence for individuals with regions of birth outside of Canada were less than that of the Canadian-born, supporting the healthy immigrant effect hypothesis and suggesting that other unmeasured factors contribute to the lower cancer incidence. This advantage appears to wane over time spent in the country and thus we also illustrate that the HIE is complex and has implications for cancer care such as the need for targeted cancer prevention tools.

Odds ratios for the incidence of any cancer, and most specific cancers were less than 1 for immigrants in reference to non-immigrants, except for stomach cancer as illustrated in Fig. 1; Table 4. Our findings are consistent with those of Bray et al. (2018), who reported that stomach cancer incidence is highest in Eastern Asian countries such as Japan and the Republic of Korea in comparison to North America. The main factors influencing stomach cancer incidence are the bacterium *Helicobacter pylori*, consumption of foods high in salt content, reduced fruit intake, increased alcohol and tobacco intake, as well as aflatoxin in crops, as demonstrated in various studies [14–18]. Recent results from the 2020 Global Cancer Observatory (GLOBOCAN) database demonstrate similar gastric cancer incidence trends with the highest age standardized incidence rates (ASIR) in Eastern Asia and Central Eastern-Europe [19]. These results suggest a possible benefit in tailoring stomach cancer prevention strategies towards individuals from East Asian countries with emphasis on dietary factors.

The second exception to the HIE phenomenon found for female non-cervical gynecological cancers also warrants further investigation. In this study, non-cervical gynecological cancers included vaginal, ovarian, and uterine cancers and female immigrants had higher odds compared to Canadian-born women (Fig. 1). Specifically, those born in European and African countries had higher odds (1.15 and 1.32), respectively compared to non-immigrants (Table 4). 2012 GLOBOCAN results showed that the highest ASIRs of ovarian cancer were present in Fiji (14.9) as well as Eastern European countries, including Latvia (14.2) and Bulgaria (14.0) with similar rates in 2020 [20, 21]. There are predisposing factors including age, family history, genetic mutations– and other studied factors such as endometriosis, high cholesterol diet and obesity that increase risk of ovarian cancer [20]. For endometrial cancer, incidence rates are greater in high-income countries with the highest levels in North America and Western Europe and lowest in South and Central Asia, and Africa. Influential factors include fewer pregnancies, late age at menopause, diet, and obesity. However, underreporting in lower-income countries is likely if cases are not diagnosed [22]. The odds of the US-born were not statistically significantly different from the Canadian-born, which is to be expected due to similar lifestyles and behaviours. Possible explanations for the higher odds observed among immigrant women born in African countries include lifestyle shifts and acculturation, such as dietary changes that may increase cancer risk. While literature shows that rates of endometrial cancer are often lower in Africa, underreporting or lack of cancer registries in low-income countries may underestimate cancer rates resulting in the appearance of higher rates in immigrants in Canada with increased screening/recording measures. Additionally, the grouping of the various non-cervical cancer types into one category may attribute to some of the variations in our results when compared to other literature. Further study with stratification by specific gynecological cancer sub-types may provide more insights.

The unweighted and unadjusted cumulative incidence curves shown in Supplementary Fig. 1 potentially demonstrates the impact of time spent in Canada since immigration on cancer incidence. However, the trends demonstrated may be impacted by other factors such as acculturation, changes in environmental exposures and aging that are not adjusted for in this figure. Studies investigating secular trends of cancer incidence in Canada have demonstrated that over the past few decades, there has been a decrease in lung, cervical, and prostate cancer incidences likely due to increased implementation and access of screening and prevention programs. However, kidney, uterine and pancreas cancers are on the rise, especially in younger adults, likely due to changes in lifestyle and prevalence of obesity conveying that incidence in immigrants and non-immigrants are shaped by multiple factors [23, 24]. The Cox regression results in Table 4 provide weighted and adjusted values, further supporting the healthy immigrant effect as shown in the increased hazard ratios based on time spent in Canada. Immigrants in Canada for 20 years or more had a hazard ratio of 0.98, which was not significantly different from the Canadian-born cohort (95% CI [0.90–1.07, *p* = 0.632). This suggests that after a period spent in Canada, the hazard of cancer incidence converges to that of non-immigrants as demonstrated in previous studies [4, 5]. Additionally, the hazard ratios for those born in the US and Europe were similar to that of the Canadian-born reference group whereas those born in South Asian or Central and South American countries had lower risk of cancer incidence at any particular time. These results are aligned with previous studies where countries with higher GDP have been associated with increased life expectancy and therefore cancer risk, as well as the possibility of regular screening resulting in greater detection [4, 25]. Additionally, immigrants born in the US and Europe may arrive in Canada with certain environmental, genetic, and lifestyle factors from their countries of birth that influence their health outcomes, such as diet or occupational exposures. These factors may contribute to the absence of the HIE among these groups. Upon prolonged exposure to the risk factors in Canada, their health outcomes may converge with the Canadian-born. Moreover, immigrants from these countries may also share similarities in health profiles upon arrival with the Canadian-born due to similar cultural and health behaviours, a possible explanation for the similar hazard ratios of cancer incidence [26].

As age, language, income, and education were controlled for, other factors such as increased use healthcare services may contribute to the convergence of the hazard rate to that of the Canadian-born cohort as immigrants become more familiar and comfortable with the healthcare system. Increased access to healthcare providers and utilization of healthcare resources and screening may lead to higher diagnoses [27, 28]. Additionally, adoption of the host country’s lifestyle and habits over time or differences in other unmeasured variables including diet, lifestyle factors and environmental exposures could also influence the HIE. Further studies should investigate the possibility that reduced access and utilization of screening among new immigrants could contribute to a perceived healthy immigrant effect, potentially masking true health outcomes. A possible explanation is that lack of knowledge of Canada’s official languages results in reduced utilization of screening tools and consequently cancer detection as shown by the hazard ratio in Table 3.

This study should be interpreted in the context of its strengths and limitations. The immigrant status variable was derived from region of birth. However, time spent in Canada and other countries may differ between individuals, impacting the adoption of habits and cultural practices from those countries. Thus, using the immigrant status variable as an indicator of immigration does not fully encapsulate the lived experiences of immigrants and their differing exposures to cancer-related risk factors. Moreover, the decision was made to exclude non-permanent residents from the study to avoid additional variability, as our focus was on immigrant health outcomes. However, we acknowledge the potential for selection bias associated with this exclusion. Additionally, while geographic regions in Canada were not included in our models as confounders, the sociodemographic variables often associated with geographical areas were included in the models. Supplementary Fig. 1 presents an unadjusted and unweighted visual of cancer incidence and should be interpreted with caution, specifically due to differences in age across the groups. For future studies, the average age in 1991 for each of the groups should be obtained to better understand the impact of time spent in Canada on cancer incidence. However, the figure provides a visual overview of potential incidence trends that are investigated further in the controlled Cox regression model. The Census also only captures information at one point in time, and thus, changes in variables such as income and education over the follow-up period were not captured. At the time of the data linkage, the CCR did not capture cancer incidence data from Quebec after 2010, which leads to the issue of under-reporting of cancer cases from that province. However, the large sample size and usage of sampling and bootstrap weights allowed us to make inferences about the Canadian population. By using CanCHEC data, and linking data from the CCR and CVSD, demographic and cancer-specific information was gathered to observe trends in cancer incidence over a 23-year period. Additionally, cancer incidence data have a good degree of accuracy due to data validation and verification procedures.

## Conclusions

Our study demonstrated a perceived healthy immigrant effect over a 23-year period where immigrants had lower odds of cancer incidence in comparison to the Canadian-born. This effect is attenuated with time spent in Canada. These findings are consistent with previous literature but also demonstrate the complexity of the effect as it depends on the type of cancer. Cancer screening and prevention initiatives tailored to higher-risk immigrant groups, with focus on specific risks and behaviours such as dietary modifications could help reduce the cancer burden in Canada. Further study into the different types of immigrants (e.g. economic), geographical regions of residence in Canada, as well as cancer mortality outcomes may provide a more all-encompassing understanding as well. To further understand this complex phenomenon and why the advantage wanes over time, individual-level health behaviours should be examined, such as diet, lifestyle, environmental exposures, and access and utilization of health care services. Access and utilization may be a major driver of this effect and deserves particular investigation to ensure equitable healthcare and outcomes.

## Electronic supplementary material

Below is the link to the electronic supplementary material.

Supplementary Material 1

## Data Availability

Data are not shareable outside the RDC. If required, data can be accessed from within an RDC. Please contact Hadassah Abraham (hadassah.abraham@bccancer.bc.ca) for further information regarding data access.

## Abbreviations

ASR: Age-standardized rate
HIE: Healthy immigrant effect
CanCHEC: Canadian census health and environment cohort
CCR: Canadian cancer registry
CVSD: Canadian vital statistics database
UBC: University of British Columbia
WESP: World economic situation and prospects
ICD: International classification of diseases
SEER: Surveillance, epidemiology, and end results
GLOBOCAN: Global cancer observatory
US: United States
