# Supplementary material for: Population-based differences in cancer incidence between immigrants and non-immigrants in Canada between 1992 and 2015
Source: BMC Public Health. 2025 May 19;25:1849. doi: 10.1186/s12889-025-23117-0 (PMC12087223; doi:10.1186/s12889-025-23117-0)
Supplement: Supplementary file 1 — Supplementary Material 1 [file 12889_2025_23117_MOESM1_ESM.docx]

**Additional File 1: Supplementary Figure 1**


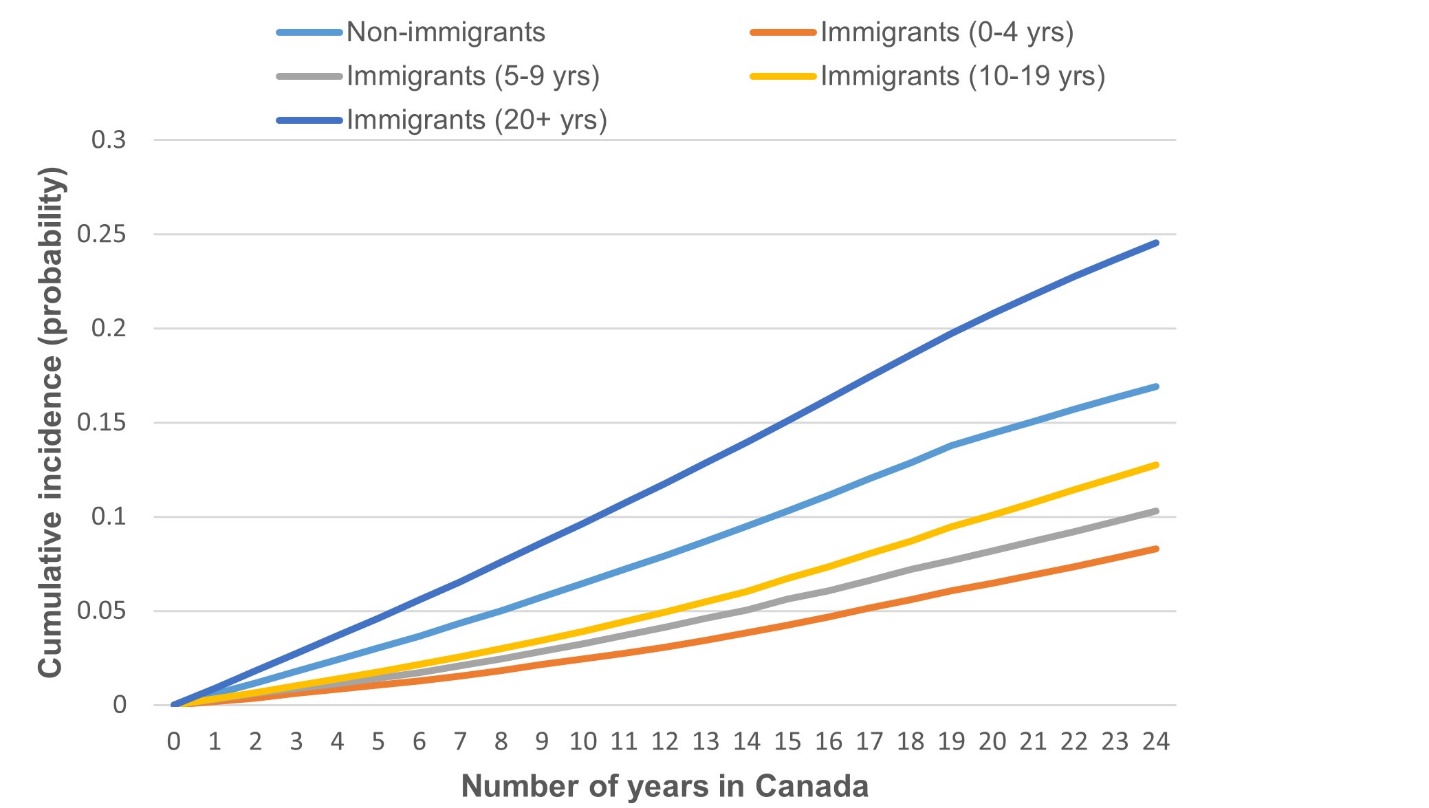


**Supplementary Figure 1:** Cumulative cancer incidence curves for non-immigrants and immigrants over the study period
